# Investigation of the Damping Capacity of CFRP Raft Frames

**DOI:** 10.3390/ma15020653

**Published:** 2022-01-15

**Authors:** Jinguang Zhang, Jun Rao, Lei Ma, Xianglong Wen

**Affiliations:** 1 School of Mechanical and Electronic Engineering, Wuhan University of Technology, Wuhan 430070, China; jgzhang@whut.edu.cn (J.Z.); rao20211214@163.com (J.R.); 13733494955@163.com (L.M.); 2 Institute of Advanced Material and Manufacturing Technology, Wuhan University of Technology, Wuhan 430070, China; 3 Hubei Provincial Engineering Technology Research Center for Magnetic Suspension, Wuhan 430070, China

**Keywords:** CFRP raft frame, damping ratio, layups, damping performance

## Abstract

In this paper, based on the composite laminated plate theory and a strain energy model, the damping capacity of a Carbon Fiber Reinforced Plastics (CFRP) raft frame was studied. According to the finite element analysis (FEA) and damping ratio prediction model, the influences of different layups on the damping capacity of the raft frame and its components (top/bottom plate and I-support) were discussed. Comparing the FEA results with the test results, it can be figured out that the CFRP laminate layup has a great influence on the damping ratio of the raft frame, and the maximum error of the first-order natural frequency and damping ratio of the top/bottom plate were 5.6% and 15.1%, respectively. The maximum error of the first-order natural frequency of the I-support between the FEA result and the test result was 7.5%, suggesting that because of the stress concentration, the error of the damping ratio was relatively large. As for the raft frame, the damping performance was affected by the I-support arrangement and the simulation analysis was in good agreement with the experimental results. This study can provide a useful reference for improving the damping performance of CFRP raft frames.

## 1. Introduction

Mechanical equipment will inevitably produce vibrations while working, and the deleterious vibrations can be reduced by installing a raft frame vibration isolator. Raft frames have outstanding vibration isolation performance, and numerous studies have been carried out to improve the damping performance of the basic raft frame structure, such as changing the geometric size or using different materials [1,2,3].

Compared with conventional metal materials, carbon fiber reinforced plastics (CFRP) have many advantages such as high specific modulus, high damping capability, high strength, and strong designability. Their damping loss factor is 1–2 orders of magnitude higher than that of metal materials [4,5]. In recent year, CFRP has been applied to design raft frames, and already been used in a wide range of fields, including satellite, spaceship, and submarine manufacture [6,7]. According to these studies [8,9], it is fairly easy to appreciate that CFRP raft frames have already been adopted successfully to isolate vibrations.

Current research on damping of composite materials mainly focuses on the variation of the microstructure features of a single laminated plate, such as its fiber volume fraction, fiber orientation, elastic modulus and aspect ratio. Related studies indicate that these factors influence the longitudinal shear damping of composite materials [10,11,12]. Macroscopically, the fiber layering angle and layup influence the damping performance, and four layups with good damping performance have been studied [13]. The lower the fiber volume fraction and the greater the fiber laying angle, the better the damping performance of the resulting composite laminates [14,15,16,17].

However, there are few studies concerned with the influence of stiffness changes caused by different layups and structures on the damping performance. Most of the research objects are still laminated plate structures. The damping performance of the more complex CFRP raft structure needs to be further studied.

Therefore, in this paper a CFRP raft frame structure was designed to investigate the effect of different factors on the final damping performance. We first calculated the damping ratio of the raft frame by theoretical models, analyzing afterwards the influence of CFRP raft frame parameters on the vibration isolation performance with the finite element simulation method, and then establishing a vibration isolation test platform for the raft frame to test its performance.

## 2. Damping Ratio

According to the concept of energy dissipation, the damping capacity of a structure is defined as the ratio of the total dissipated energy to the maximum strain energy during a vibration period of the system [18,19]:

(1) $$\zeta =2\pi \eta =\frac{\Delta U}{U}$$

where ζ is damping ratio, η is damping loss factor, ΔU and U represent the dissipated energy and the total strain energy stored in a vibration period, respectively.

A composite laminate is anisotropic, and the damping loss factor of the structure can be expressed as follows:

(2) $$\eta =\frac{\sum_{k=1}^{n}\eta_{ij}U_{ij}^{k}}{\sum_{k=1}^{n}U_{ij}^{k}}$$

where $U_{ij}^{k}$ is the sum of the strain energy of the *k*^th^ cell of the composite structure generated by the stress $\sigma_{ij}$ of the layer, $\eta_{ij}$ is the damping loss factor in the corresponding direction, 1 refers to the positive axis direction, 2 refers to the direction perpendicular to the fiber, and 3 refers to the thickness direction. Under the small deformation assumption and the linear elasticity assumption, the strain energy generated by each unit can be calculated using Equation (3):

(3) $$U_{ij}^{k}=0.5\int^{\;}\sigma_{ij}^{k}\varepsilon_{ij}^{k}dV^{k}\left(i,j=1,\;2,\;3\right)$$

$\sigma_{ij}^{k},$ $\varepsilon_{ij}^{k}\;\left(i,\;j=1,\;2,\;3\right)$ represent the stress and strain components in the *k^th^* unit of the composite laminate, respectively. $V^{k}$ is the integral volume of unit *k*.

The raft frame is composed of many parts, the proportion of strain energy loss of diverse parts are different, so we propose the definition of strain energy loss in different directions for each part of the structure:

(4) $$SE_{ij}^{p}=100\frac{\Delta U_{ij}^{p}}{max\left\{\Delta U_{total}^{1},\Delta U_{total}^{2}\ldots \ldots ,\Delta U_{total}^{n}\right\}}$$

$U_{total}^{n}$ represents the sum of strain energy loss, $\Delta U_{ij}^{p}$ is the strain energy loss generated by the stress $\sigma_{ij}$ in the part *p*. $SE_{ij}^{p}$ represent the proportion of strain energy loss generated by stress $\sigma_{ij}$ in the part *p* of the structure. The component with the largest strain energy loss in the structure is:

(5) $$SE^{p_{max\;}}\;=\sum_{i=1}^{3}\sum_{j=1}^{3}SE_{ij}^{p}=100\left(i,j=1,\;2,\;3\right)$$

For the whole laminate structure, combined with Equation (1), during a vibration period, total dissipated energy and total strain energy can be expressed as:

(6) $$\Delta U=\sum_{k=1}^{n}\sum_{i=1}^{3}\sum_{j=1}^{3}2\pi \eta_{ij}U_{ij}^{k}2\pi \sum_{k=1}^{n}\sum_{i=1}^{3}\sum_{j=1}^{3}\frac{1}{2}\int^{\;}\eta_{ij}\sigma_{ij}^{k}\varepsilon_{ij}^{k}dV^{k}$$

(7) $$U=\sum_{k=1}^{n}\sum_{i=1}^{3}\sum_{j=1}^{3}U_{ij}^{k}=\sum_{k=1}^{n}\sum_{i=1}^{3}\sum_{j=1}^{3}\frac{1}{2}\int^{\;}\sigma_{ij}^{k}\varepsilon_{ij}^{k}dV^{k}\left(i,j=1,\;2,\;3\right)$$

The damping loss factor of the structure is:

(8) $$\eta =\frac{\Delta U}{2\pi U_{max}}=\frac{2\pi \sum_{k=1}^{n}\sum_{i=1}^{3}\sum_{j=1}^{3}\frac{1}{2}\int^{\;}\eta_{ij}\sigma_{ij}^{k}\varepsilon_{ij}^{k}dV^{k}}{2\pi \sum_{k=1}^{n}\sum_{i=1}^{3}\sum_{j=1}^{3}\frac{1}{2}\int^{\;}\sigma_{ij}^{k}\varepsilon_{ij}^{k}dV^{k}}\;=\frac{\sum_{k=1}^{n}\sum_{i=1}^{3}\sum_{j=1}^{3}\int^{\;}\eta_{ij}\sigma_{ij}^{k}\varepsilon_{ij}^{k}dV^{k}}{\sum_{k=1}^{n}\sum_{i=1}^{3}\sum_{j=1}^{3}\int^{\;}\sigma_{ij}^{k}\varepsilon_{ij}^{k}dV^{k}}$$

**Figure 1:**
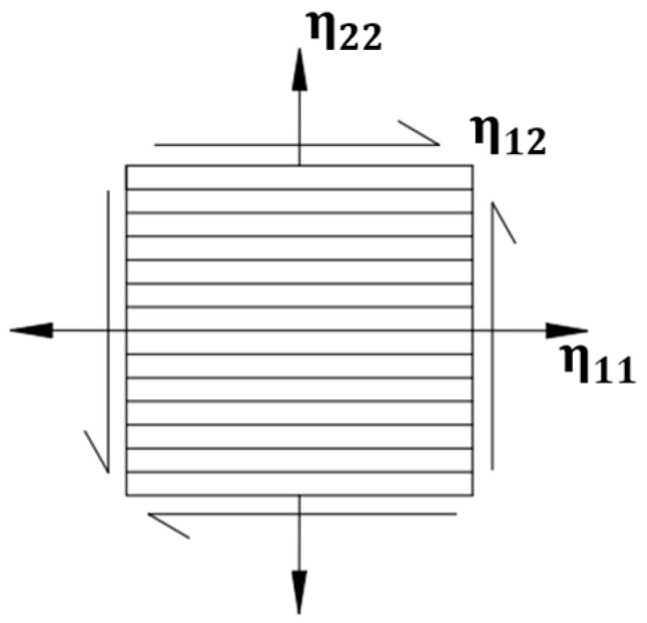
Direction of damping loss factors of composite laminated plate.

The unidirectional prepreg T700/YPH-42T consists of 68% T700 carbon fibers and 32% YPH-42T epoxy resin and the thickness of one layer is 0.2 mm. The material properties are listed in Table 1 [20]. There are six damping loss factors in six directions of the composite material, where direction 1 is the fiber direction, direction 2 and 3 indicate the transverse direction. As for the CFRP laminated plate, only three damping loss factors are considered, the directions are shown in Figure 1. The damping loss factors in three direction of the laminated plate are as follows: $\eta_{11}=0.82\%$ $\eta_{22}=2.98\%$, $\eta_{12}=8.57\%$ [21,22].

The damping loss factor of the structure is converted into the damping ratio [6]:

(9) $$\zeta \;=\frac{\eta }{\sqrt{4+\eta^{2}}}\;$$

where ζ is the structural damping ratio.

## 3. Simulation

### 3.1. Model

The software ABAQUS (Dassault SIMULIA, Johnston, RI, USA.) is adopted for the FEA of the raft frame, the continuum shell SC8R elements are applied and the sweep meshing method is adopted because of the directivity of composite materials. In ABAQUS, the analysis models have been simplified, and the connection between different parts is “Tie”.

**Figure 2:**
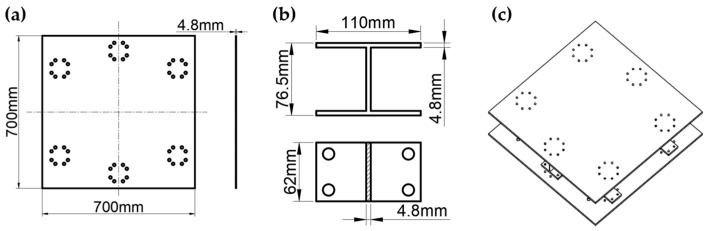
(**a**) Top/bottom plates of the raft frame. (**b**) I-support. (**c**) I-support raft frame.

**Figure 3:**
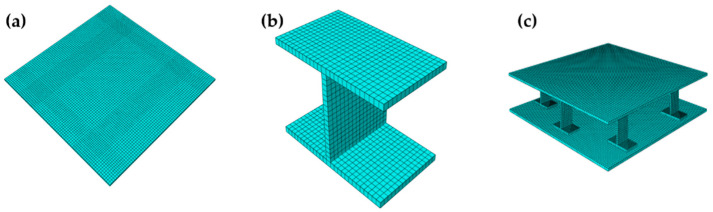
Simulation model: (**a**) Top/bottom plate of the raft frame. (**b**) I-support. (**c**) I-support raft frame.

**Figure 4:**
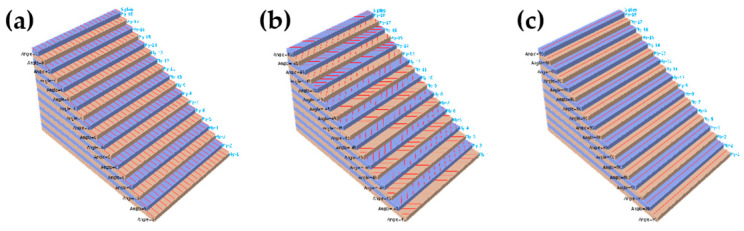
Three main layups (Fiber orientation): (**a**) C0. (**b**) C45. (**c**) C90.

The components of the raft frame are shown in Figure 2. The simulation model and fiber orientation are shown in Figure 3 and Figure 4, respectively.

**Figure 5:**
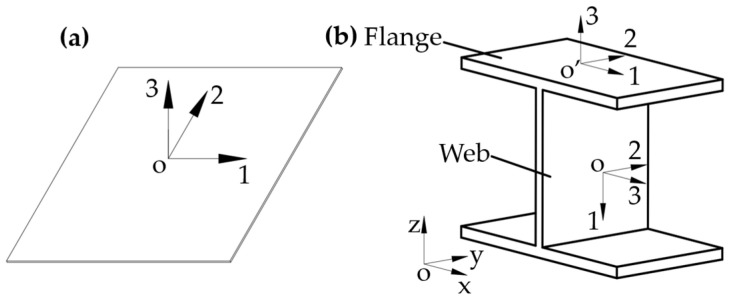
Layer coordinate systems of different components: (**a**) Top/bottom plate. (**b**) I-support.

Seven layups are set between 0° to 90° at 15° intervals, denoted by C0, C15, C30, C45, C60, C75, and C90. The bending deformation appears while the raft frame is being excited. The regularized stiffness coefficients (D_11_*, D_22_* and D_66_*) could be calculated to describe the stiffness change of the laminates [23]. The data are shown in Table 2, Figure 5 shows the layer coordinate system on each component. Directions 1, 2 and 3 represent the main stiffness direction, the secondary stiffness direction and the thickness direction of the structure in the layer coordinate system, respectively.

The stress and strain values of non-rigid body modes are exported by using software ABAQUS, and the damping ratio of different modes can be calculated by the MATLAB program (MathWorks, Natick, MA, USA) [20].

### 3.2. Simulation Analysis

During the simulation, the free modal is analyzed, the first six orders are rigid body modes, and the seventh order is non-rigid mode. In this paper, only non-rigid modes are considered.

#### 3.2.1. Simulation Analysis of the Top/Bottom Plate

**Figure 6:**
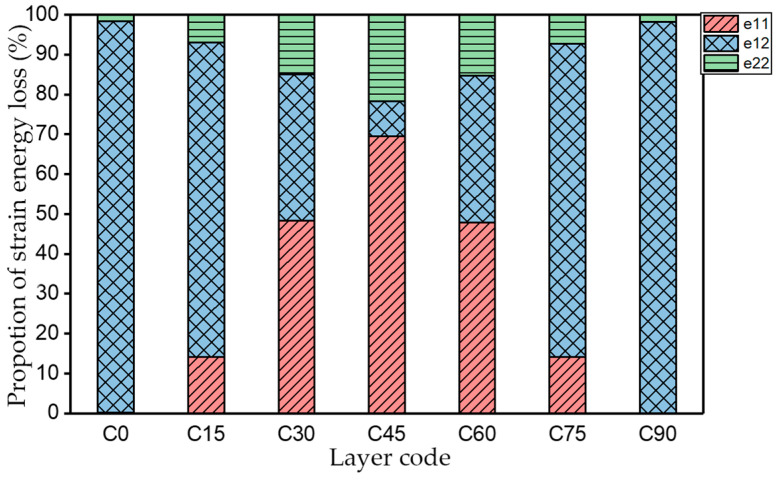
Proportion of strain energy loss in each direction of first-order torsional modal shape.

Table 3 shows the first four orders modal shape of the top/bottom plate. The natural frequency and damping ratio of first-order torsional modal shape are shown in Table 4, the proportion of strain energy loss in different directions are calculated according to Equation (4), as shown in Figure 6 (e11, e12 and e22 represent the three directions of the coordinate system).

**Figure 7:**
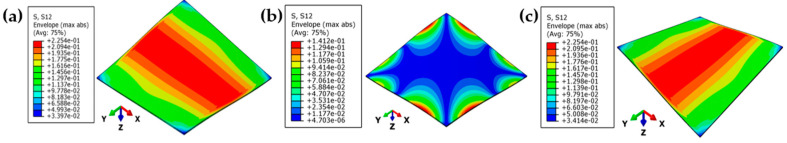
Stress distribution of first-order torsional modal shape in 12 direction: (**a**) C0. (**b**) C45. (**c**) C90.

**Figure 8:**
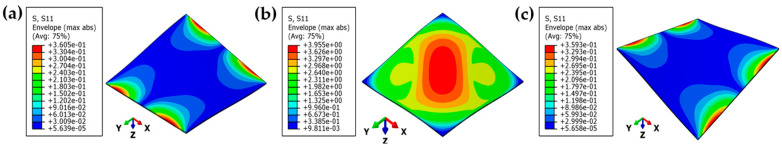
Stress distribution of first-order torsional modal shape in 11 direction: (**a**) C0. (**b**) C45. (**c**) C90.

As shown in Table 4 and Figure 6, from C0 to C90 of the plate, the proportion of strain energy loss in each direction of first-order modal shape increases first, reaching its peak at C45, and then decreases. Combined with the derivation process of the internal force of laminated plate, it is seen that torsional deformation is a macroscopic phenomenon caused by in-plane shear stress, therefore, the natural frequency of the torsional modal shape is mainly affected by the torsional stiffness coefficient D_66_*. The natural frequency increases correspondingly when D_66_* increases from C0 to C45 gradually. In layer C0 and C90, deformation direction is 45 degrees to the fiber orientation (X/Y direction). The shear deformation reaches its maximum, therefore, strain energy loss mainly concentrated in the 12 direction, as shown in Figure 7. In layer C45, the fiber orientation is the same as the deformation direction, the strain energy loss reaches its maximum in 11 direction, as shown in Figure 8. The damping loss factor is small relatively in 11 direction, in this modal shape, the damping ratio decreases gradually from layer C0 to layer C45.

**Figure 9:**
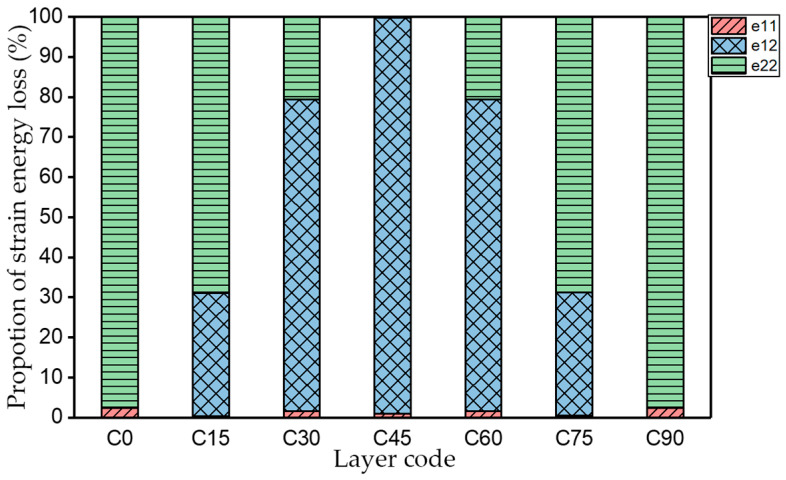
Proportion of strain energy loss in each direction of first-order bent modal shape.

The natural frequency and damping ratio of first-order bent modal shape are shown in Table 5, and strain energy loss is shown in Figure 9.

**Figure 10:**
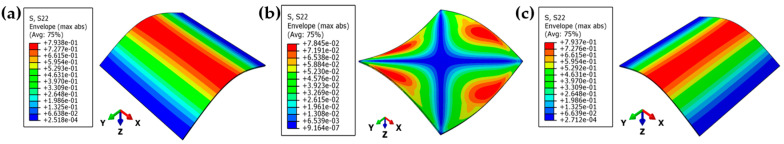
Stress distribution of first-order bent modal shape in 22 direction: (**a**) C0. (**b**) C45. (**c**) C90.

**Figure 11:**
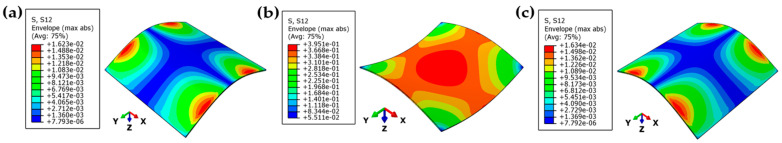
Stress distribution of first-order bent modal shape in 12 direction: (**a**) C0. (**b**) C45. (**c**) C90.

As shown in Table 5 and Figure 9, the trend of natural frequency of first-order bent modal shape is consistent with the damping ratio, which increases from C0 to C45 and gradually decreases from C45 to C90. The layups (C45) with smaller bending stiffness factor bends first and the natural frequency also increases. In layer C0 and C90, the direction of bending deformation is perpendicular to the fiber orientation (X/Y direction), therefore, the strain energy loss is mainly concentrated in direction 22, as shown in Figure 10. In layer C45, the angle between deformation direction and fiber direction is 45 degrees, and the shear deformation is at its maximum, so the strain energy loss in 12 direction reaches its peak, as shown in Figure 11. Mandal et al. [24] calculated the damping loss factors of rectangular laminates by the half-power method, and their result shows that the damping loss factor increases with the rising of flexural stiffness.

#### 3.2.2. Simulation Analysis of I-Support

**Figure 12:**
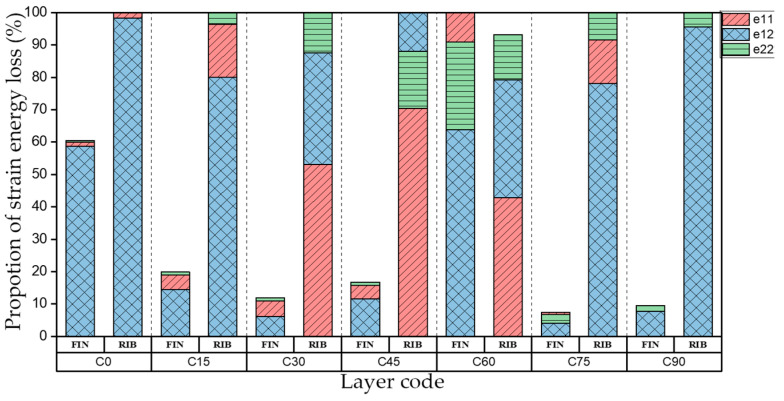
Proportion of strain energy loss in different direction of the torsional modal shape of the web plate.

Table 6 shows the first four orders modal shape of I-support. The natural frequency and damping ratio of torsional modal shape of the web plate of I-support are shown in Table 7. Figure 12 indicates the strain energy loss in different directions. FIN represents the flange plate and RIB represents the web plate of the I-support.

As shown in Table 7 and Figure 12, in the torsional modal shape, natural frequency is consistent with the variation trend of torsional stiffness coefficient D_66_* in the laying coordinate system of the web plate while the fiber layering angle increases. The strain energy loss in different directions of RIB is consistent with the first-order torsional modal shape of the plates. The strain energy loss of FIN increases obviously at layer C60, and the flange plates are bent at the same time, the distribution of strain energy loss of the flange plate is consistent with the plates under the first-order bent modal shape at layer C60. Compared with layer C30, the damping ratio of layer C60 improved obviously.

**Figure 13:**
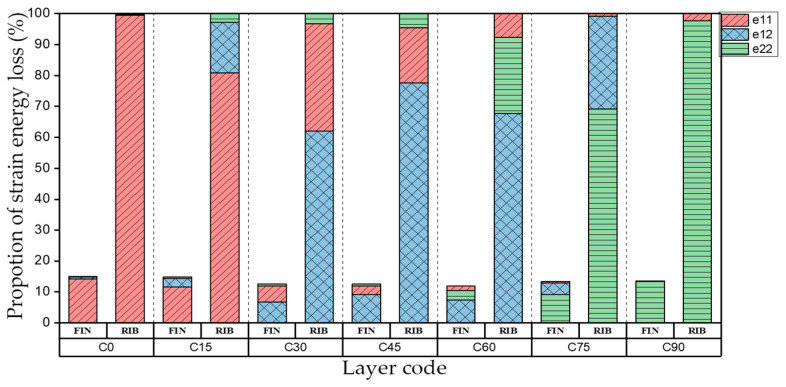
Proportion of strain energy loss in different directions of the bent modal shape of the web plate.

The natural frequency and damping ratio of the bent modal shape of the web plate of I-support are shown in Table 8, Figure 13 shows the strain energy loss in different directions.

Table 8 shows that when the fiber layering angle increases, the natural frequency decreases gradually. This happens because the bending stiffness coefficient D_11_* decreases in the layer coordinate system of the web plate. As shown in Figure 13, web plates contribute to most of the strain energy loss, and the strain energy loss in different direction is also consistent with the first-order bent modal shape of the top/bottom plate.

**Figure 14:**
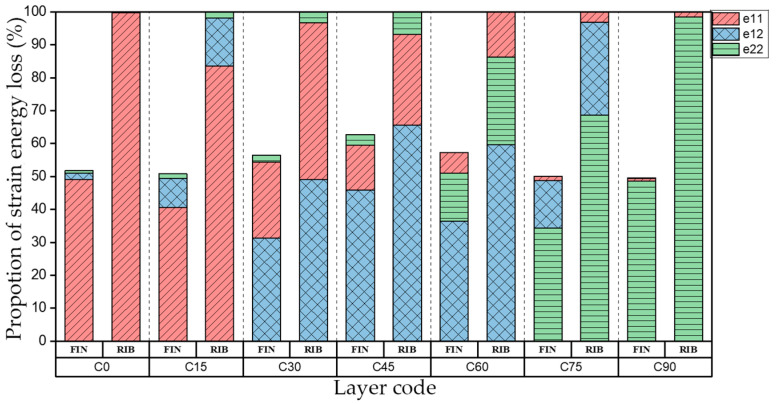
Proportion of strain energy loss in different direction of bent modal shape of the flange plates.

Table 9 shows the natural frequency and damping ratio of bent modal shape of the flange plate of I-support, and Figure 14 shows the strain energy loss in different directions.

The natural frequency diminishes with the decrease of the bent stiffness coefficient D_11_* in the flange and web laying coordinate system when the flange plates and web plates are bent. Figure 14 shows that the trend of strain energy loss ratio of the flange and web plates are almost in accordance.

**Figure 15:**
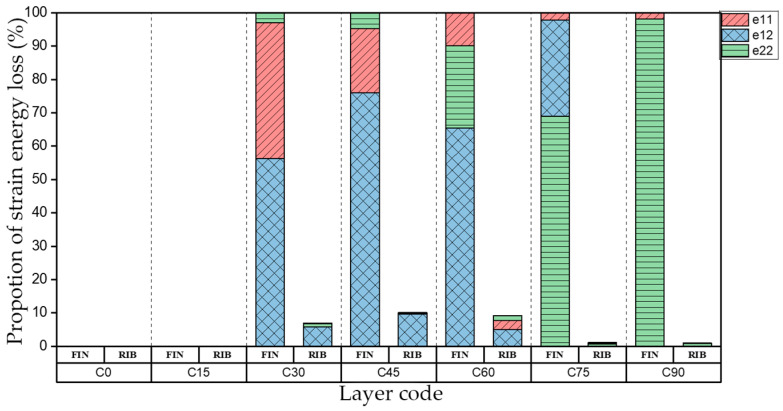
Proportion of strain energy loss in different directions of reversed bent modal shape of the flange plates.

The natural frequency and damping ratio when the flange plates of I-support undergo reversed bending are shown in Table 10 and the strain energy loss of different directions is shown in Figure 15.

The modal shapes of C0 and C15 are different from the others, so the flange plate is not included in the comparison. It can be obtained from Table 10 and Figure 15 that in the bent modal shape of the flange plate, the natural frequency of I-supports diminishes with the decrease of the bent stiffness coefficient D_11_* in the laying coordinate system. The strain energy loss is contributed to the flange plates, so the layups of the flange plate can be adjusted between C45 and C90 to obtain better damping capacity.

**Figure 16:**
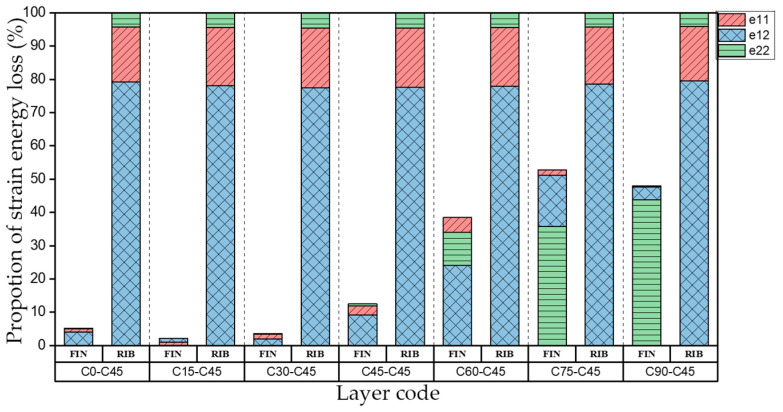
Proportion of strain energy loss in bent modal shape of I-support.

In order to investigate the influence of flange layer change on the web plate. Web plates are set as C45, N indicates the flange plate and set as layer C0~C90. The layup of I-support can be described as N-C45. The strain energy loss of different directions is shown in Figure 16.

When the fiber layering angle of the flange plates increases, the proportion of strain energy loss of the support increases gradually, and the proportion of strain energy loss in different directions is various under different layups; the proportion of strain energy loss of web plate decreases in the meanwhile. However, the proportion of strain energy loss in each direction remains constant under different layups of the flange plates, which shows that the change of layer mainly affects the proportion of strain energy loss distribution. That is, the damping capacity of laminates is determined by the fiber layering angle, the fiber layering angle of the flange plate can be adjusted to dissipate more energy.

The first four modal shape of the flange plates and web plates are bent, the stiffness caused by the change of layups has a great influence on the natural frequency. The strain energy loss distributions of the flanges and webs in different direction are consistent with those of the independent laminates in corresponding modes, the fiber layering angle determines the damping capacity of the laminates under bending deformation. The stiffness of the laminates with different layups affects the damping performance of the structure, so the lamination can be adjusted to modify the strain energy loss ratio of specific laminates.

#### 3.2.3. Simulation Analysis of CFRP Raft Frame

Selecting the layups C0, C45 and C90 to represent the stiffness distribution and damping distribution trend. As for the I-support, the peak value of damping ratio appears at C60, and the maximum and minimum bending Ds D_11_* are in C0 and C90 respectively. Therefore, the selection of group C0, C60 and C90 can represent the trend of the stiffness distribution and damping distribution.

The damping ratios are calculated according to the nine groups of CFRP raft frame in Table 11. The layups of top plate -I-support-bottom plate are represented by CX-CX-CX, respectively.

**Figure 17:**
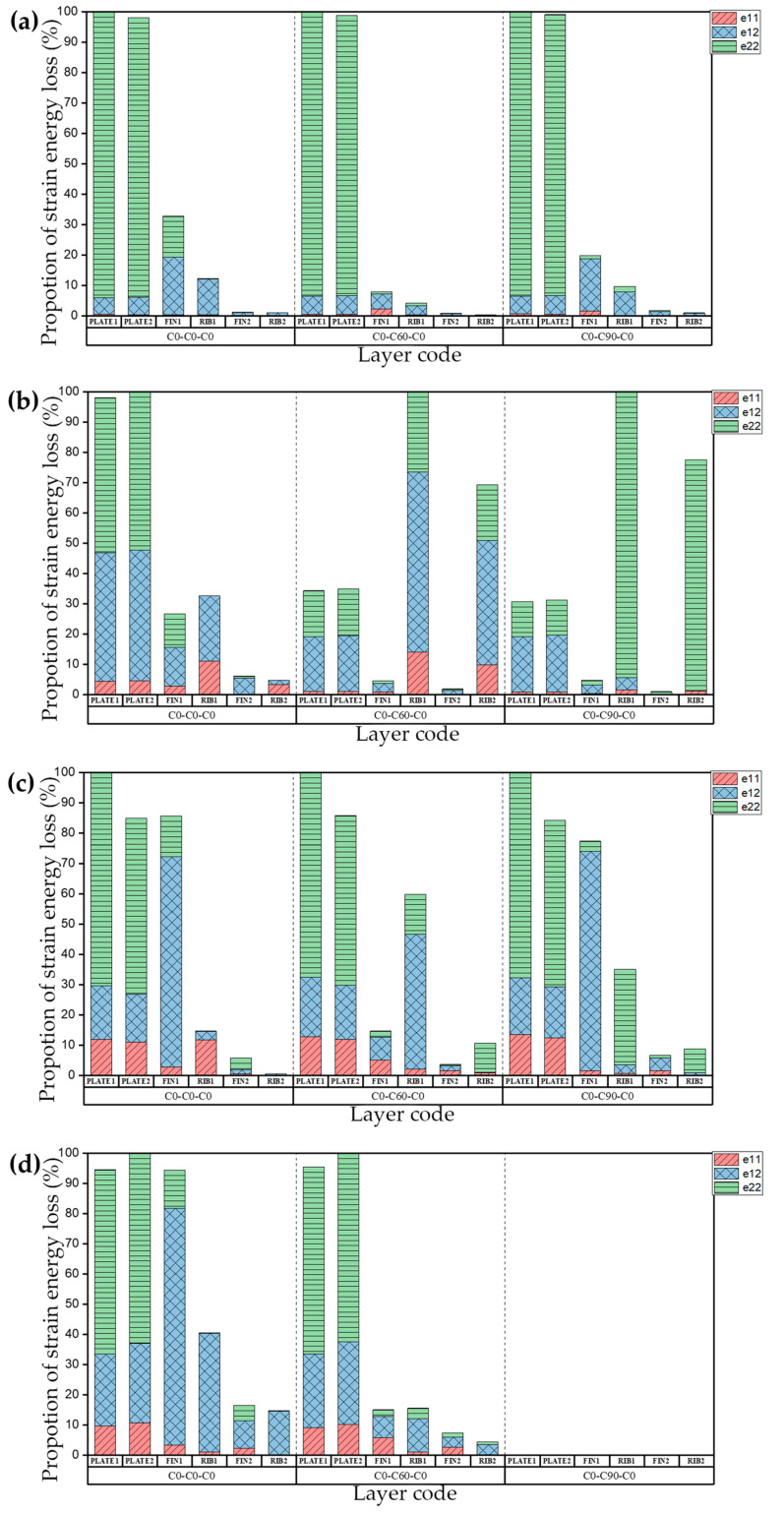
Proportion of strain energy loss in the first four orders of the raft frame with layups C0-N-C0: (**a**) First order. (**b**) Second order. (**c**) Third order. (**d**) Fourth order.

The natural frequency and damping ratio of the raft frame with layup C0-N-C0 are shown in Table 12, Figure 17 shows the strain energy loss. PLATE1, PLATE2, FIN1, RIB1, FIN2, RIB2 represent the top plate, bottom plate, flange plate, web plate, axial flange plate, axial web plate, respectively.

**Figure 18:**
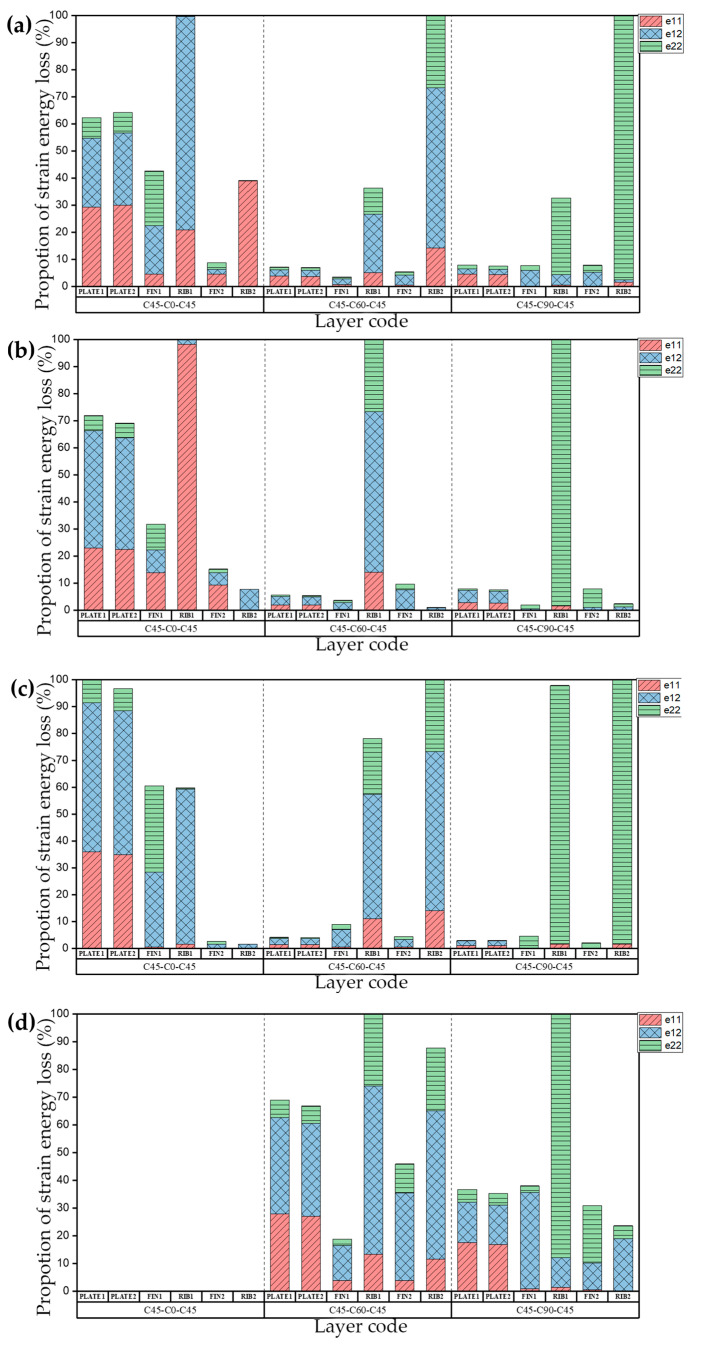
Proportion of strain energy loss in the first four orders of the raft frame with layup C45-N-C45: (**a**) First order. (**b**) Second order. (**c**) Third order. (**d**) Fourth order.

Table 13 shows the natural frequency and damping ratio of the raft frame with layup C45-N-C45, and Figure 18 shows the strain energy loss.

**Figure 19:**
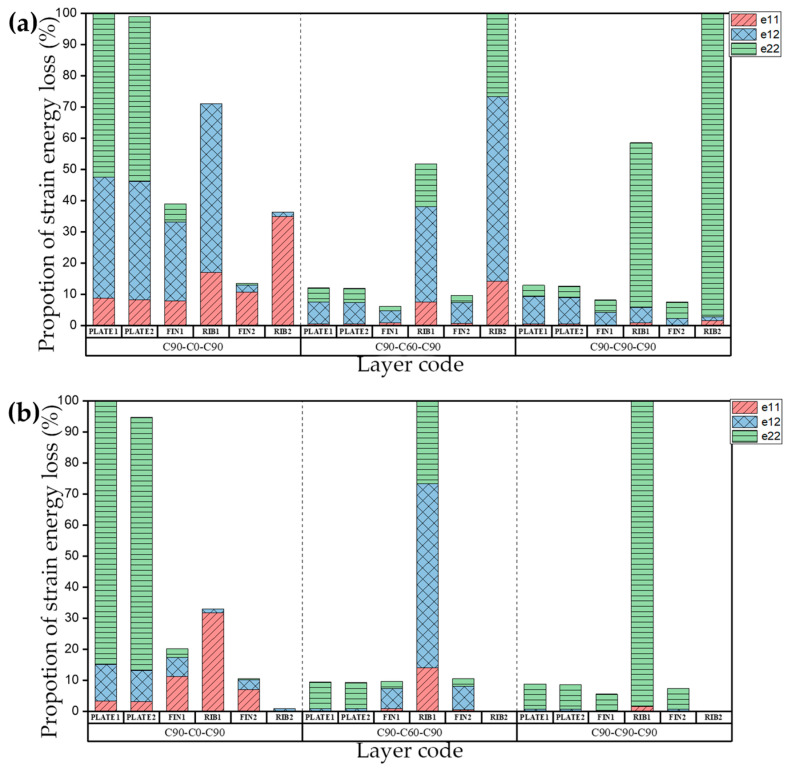

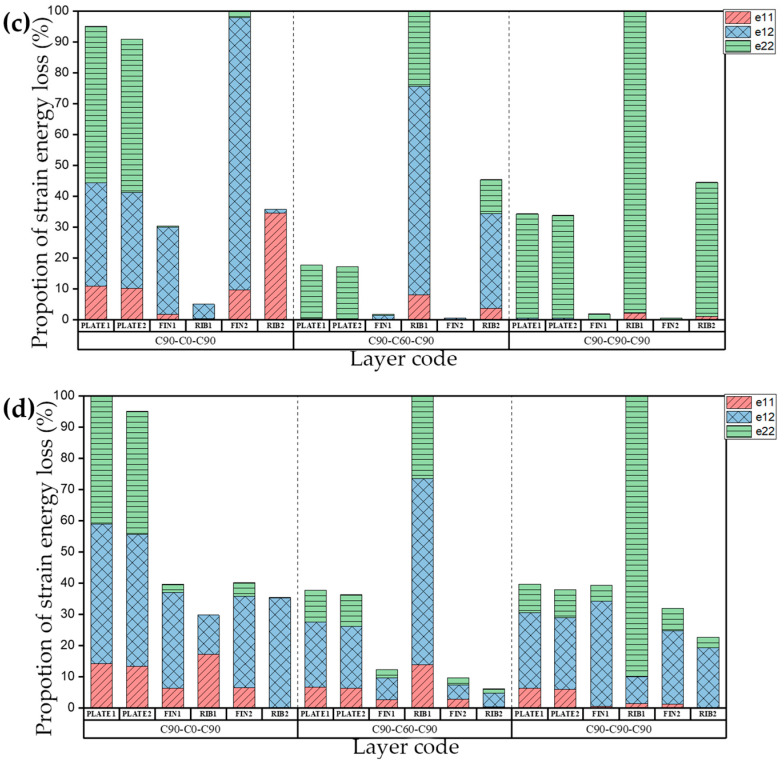
Proportion of strain energy loss in the first four orders of the raft frame with layup C90-N-C90: (**a**) First order. (**b**) Second order. (**c**) Third order. (**d**) Fourth order.

Table 14 shows the natural frequency and damping ratio of the raft frame with layup C90-N-C90, and Figure 19 shows the strain energy loss.

We can draw the conclusion that if the layups of the plates of raft frame lead to unbalanced stiffness, the influence of the layups of the I-support on the natural frequency and damping ratio of the raft frame is determined mainly by the bending coefficient, and the greater the bending coefficient of I-support, the less the natural frequency and damping ratio of the structure are affected by the layup changes. If the layups of the top/bottom plate balance the stiffness (i.e., D_11_* = D_22_*), the natural frequency of the corresponding modes are generally higher. This phenomenon indicates that the top/bottom plate itself is not prone to bending deformation, and the natural frequency and damping ratio of the raft frame are more sensitive to the change of I-support stiffness.

## 4. Experiment of the CFRP Raft Frame

### 4.1. Structure of the CFRP Raft Frame

**Figure 20:**
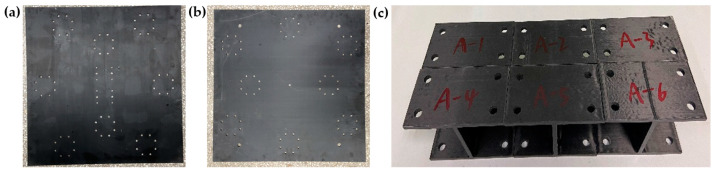
Components of the CFRP raft frame: (**a**) Top Plate. (**b**) Bottom Plate. (**c**) I-supports.

The plates of the CFRP raft frame here have uneven stiffness (D_11_* ≠ D_22_*). Table 15 shows the layups and in-plane regularized stiffness parameters. Figure 20 shows the I-support and top/bottom plates of the CFRP raft frame.

### 4.2. Modal Analysis

**Figure 21:**
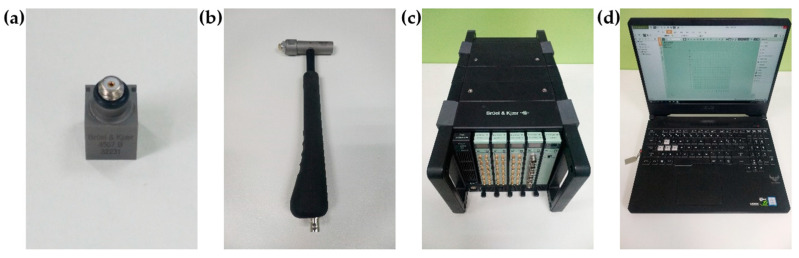
Experimental equipment: (**a**) accelerometer. (**b**) impact hammer. (**c**) data acquisition system. (**d**) computer.

**Figure 22:**
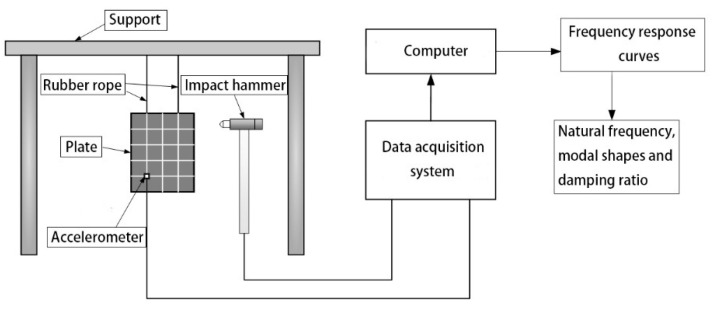
Modal testing system of the CFRP raft frame.

The modal analysis module in B&K Connect software platform (Brüel & Kjær, Copenhagen, Denmark) is applied to carry out the modal analysis experiments. The main instruments involved are accelerometers, impact hammer, data acquisition system and computer, as shown in Figure 21. In order to get the modal shape and damping ratio of different components, importing the 3D model into the computer of B&K, setting the accelerometer point and impact point as the same as the physical model, the modal testing system is shown in Figure 22.

(1) Modal test of the top/bottom plates

**Figure 23:**
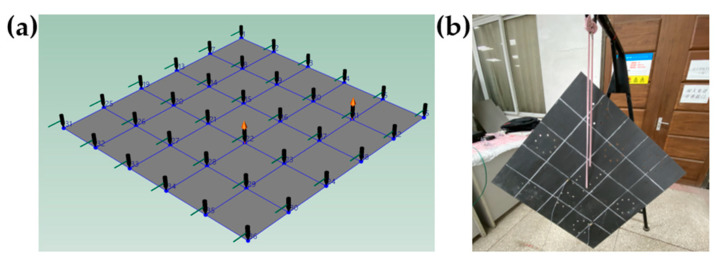
Measuring point and support of the plate. (**a**) Measuring point of the plate. (**b**) Support of the plate.

The plate of the raft frame is suspended with rubber rope to simulate the free constraint state. There are 36 black knock points and two red accelerometer measuring points, as shown in Figure 23.

According to the layups of the plates of the raft frame designed in Table 15, the simulation results can be obtained through the FEA, and the test results can be carried out by the B&K Connect software platform. Table 16 shows the comparison between the natural frequency and damping ratio of the test and simulation result of the plates.

In Table 16, compared with the test results, the maximum error of the natural frequency between the last three simulation results is 5.6%, which is consistent with the test results. The method of using rubber rope suspension to simulate free constraint results is in large error from the first order value. The error of damping ratio fluctuates around 10%, which means the simulation results are consistent with the experimental results within the margin of error.

(2) Modal test of I-support

**Figure 24:**
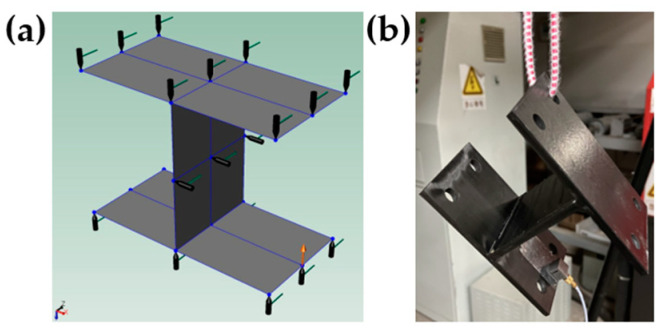
Measuring points and support of I-support: (**a**) Measuring point of I-support. (**b**) Support of I-support.

The I-support is suspended with rubber rope to simulate the free constraint state. There are 21 black knock points and one red accelerometer measuring point, as shown in Figure 24.

The DOF of the signal acquisition is parallel to the web plates, therefore, the natural frequency and damping ratio of second order were not obtained as the acceleration signal in the direction of web plates is not collected. As shown in Table 17, taking the average value of the test result and compare it with the simulation result, the simulation results of the natural frequency agree well with the test results with a maximum error of 7.5%; The error between simulation result and test result of damping ratio of first and fourth order is minor. The error of damping ratio of third order is distinct, summed up to 28.9%, according to the strain energy loss diagram of I-support, the ratio of strain energy loss between the flange plate and web plate is 1:2 while the other three orders are 1:10 in this mode, the stress of the flange plates and the web plates have great influence on each other under the corresponding condition, and the joint will also cause more strain energy loss due to stress concentration.

(3) Modal analysis of CFRP raft frame

**Figure 25:**
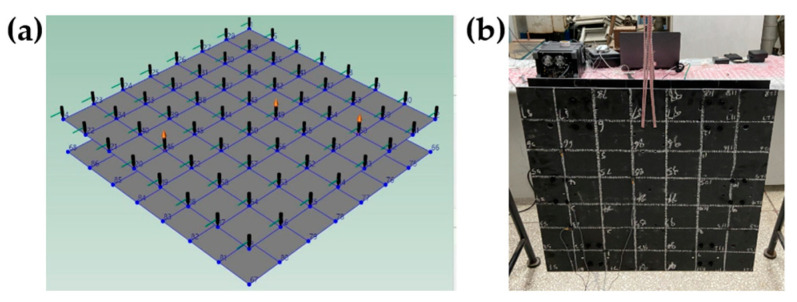
Measuring point and support of I-support raft frame: (**a**) Measuring point of I-support raft frame. (**b**) Support of I-support raft frame.

The CFRP raft frame is suspended with rubber rope to simulate free constrain state. There are 64 black knock points and three red accelerometer measuring points, as shown in Figure 25.

**Figure 26:**
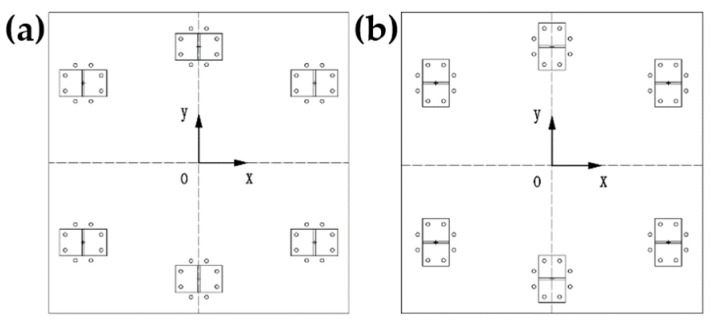
Two arrangement of I-support: (**a**) Arrangement in X direction. (**b**) Arrangement in Y direction.

Changing the installation direction of I-support to explore the influence of stiffness change on damping capacity of the raft frame, as shown in Figure 26.

As Shown in Table 18, modal shape in simulation result is consistent with the test result, the stiffness changes because the different arrangement of the I-support, this indicates that the change of stiffness influence the inherent characteristics of the structure.

Table 19 shows that the simulation values and test values of natural frequency and damping ratios of the raft frame with arrangement in X/Y direction are significantly different, the error of natural frequency ranges from 25% to 40%, as well as the damping ratio. The main reason is that both stiffness and damping have nonlinear characteristics due to bolt connection, while in software ABAQUS, the constraint “Tie” is used to connect the part, and there is no relative slip displacement and the stiffness is large, causing large results of natural frequency and damping ratio calculation.

The stiffness distribution of the structure is altered by changing the arrangement of I-supports. The maximum change of natural frequency and damping ratio are 10.1% and 43.6% in the test result, respectively. The test results show that the stiffness influence the damping capacity of complex structure obviously, and the damping capacity can be maximized by adjusting the stiffness distribution.

## 5. Conclusions

Based on the classical laminate theory, the free vibration of a CFRP raft frame and the influence of different carbon fiber prepreg layups on the damping capacity of a raft frame and its components are explored. According to the strain energy model of carbon fiber composite laminates, the damping ratio of each component have been calculated by using the MATLAB software.

(1) The natural frequency and damping ratio of the plates of the raft frame are affected by the fiber orientation, and the minimum stiffness coefficient can be increased by adjusting the fiber layering angle, which can improve the damping capacity. However, the conclusion is the opposite for torsional modal shapes.
(2) The change of stiffness caused by fiber layering angle has a significant influence on the natural frequency of the flange plate and web plate of the I-support. The damping ratio can be increased by adjusting the fiber layering angle of the layups.
(3) As for the raft frame, if the layups lead to uneven stiffness of plates, the damping capacity can be greatly influenced by the fiber layering angle; if the stiffness is balanced and generally large, the angle has a greater influence on the damping of the raft frame.
(4) Different arrangements of I-support indicate that the change of stiffness has great influence on the damping capacity and natural frequency, and the stiffness can be changed by adjusting the arrangement to optimize the damping capacity.

## Figures and Tables

**Table 1:** Material properties of CFRP T700/YPH-42T prepreg.

E_1_ (GPa)	E_2_ (GPa)	G_12_ (GPa)	$\nu_{12}$	ρ
150	9	5.12	0.24	1650

E_1_: longitudinal modulus, E_2_: transverse modulus, G_12_: shear modulus in 1–2 direction, $\nu_{12}$: Poisson’s ratio in 1–2 direction, ρ: density.

**Table 2:** Stiffness coefficient of laminates.

Layer Code	Layups	D_11_* (GPa)	D_22_* (GPa)	D_66_* (GPa)
C0	[0°]_24_	142.92	9.05	4.60
C15	[±15°]_6S_	125.97	10.04	12.59
C30	[±30°]_6S_	85.50	18.57	28.56
C45	[±45°]_6S_	44.05	44.05	36.55
C60	[±60°]_6S_	18.57	85.50	28.56
C75	[±75°]_6S_	10.04	125.97	12.59
C90	[90°]_24_	9.05	142.92	4.60

**Table 3:** The first four orders modal shape of the top/bottom plate.

Layer Code	First-Order	Second-Order	Third-Order	Fourth-Order
C0	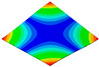	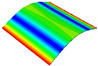	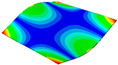	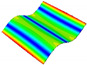
C15	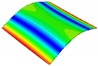	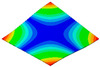	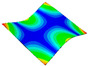	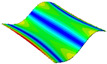
C30	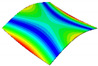	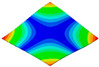	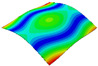	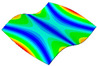
C45	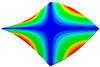	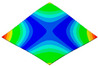	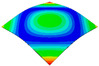	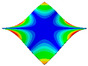
C60	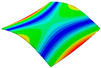	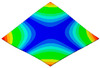	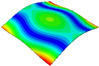	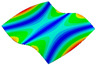
C75	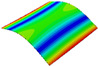	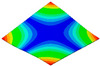	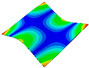	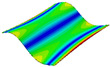
C90	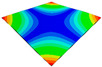	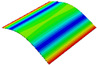	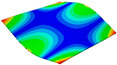	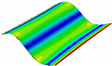

**Table 4:** Natural frequency and damping ratio of first-order torsional modal shape.

Layer Code	C0	C15	C30	C45	C60	C75	C90
Natural Frequency (Hz)	17.5	28.1	41.6	46.8	41.6	28.1	17.5
Damping Ratio (%)	4.11	1.73	0.73	0.53	0.73	1.73	4.11

**Table 5:** Natural frequency and damping ratio of first-order bent modal shape.

Layer Code	C0	C15	C30	C45	C60	C75	C90
Natural Frequency *f* (Hz)	23.9	24.1	26.0	27.5	26.0	24.1	23.9
Damping Ratio *ζ* (%)	1.59	1.891	2.77	3.91	2.78	1.89	1.61

**Table 6:** The first four order modal shape of I-support.

Layer Code	First-Order	Second-Order	Third-Order	Fourth-Order
C0	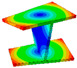	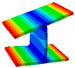	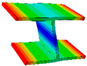	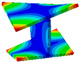
C15	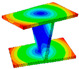	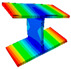	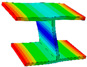	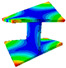
C30	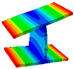	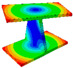	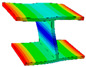	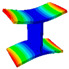
C45	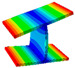	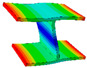	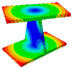	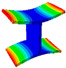
C60	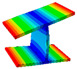	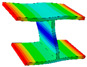	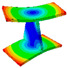	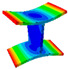
C75	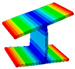	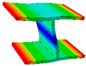	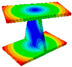	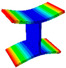
C90	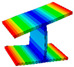	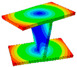	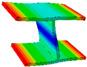	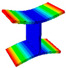

**Table 7:** Natural frequency and damping ratio of torsional modal shape of the web plate.

Layer Code	C0	C15	C30	C45	C60	C75	C90
Natural Frequency *f* (Hz)	449.2	624.3	821.4	874.2	768.5	537.8	353.2
Damping Ratio *ζ* (%)	3.62	1.58	0.70	0.58	1.08	1.76	3.89

**Table 8:** Natural frequency and damping ratio of the bent modal shape of the web plate.

Layer Code	C0	C15	C30	C45	C60	C75	C90
Natural Frequency *f* (Hz)	811.8	767.0	612.3	424.1	287.2	241.7	237.4
Damping Ratio *ζ* (%)	0.41	0.50	0.96	1.52	1.92	1.79	1.58

**Table 9:** Natural frequency and damping ratio of bent modal shape of the flange plates of I-support.

Layer Code	C0	C15	C30	C45	C60	C75	C90
Natural Frequency *f* (Hz)	1393.5	1333	1141.9	859.7	603.1	491.9	479.0
Damping Ratio *ζ* (%)	0.41	0.48	0.80	1.21	1.59	1.67	1.56

**Table 10:** Natural frequency and damping ratio of reversed bent modal shape of the flange plates.

Layer Code	C0	C15	C30	C45	C60	C75	C90
Natural Frequency *f* (Hz)	-	-	1692.1	1142.4	775.5	641.5	626.5
Damping Ratio *ζ* (%)	-	-	0.92	1.56	1.69	1.72	1.57

**Table 11:** CFRP raft frame layup combinations.

Configuration		
C0-C0-C0	C0-C60-C0	C0-C90-C0
C45-C0-C45	C45-C60-C45	C45-C90-C45
C90-C0-C90	C90-C60-C90	C90-C90-C90

**Table 12:** Natural frequency and damping ratio of the raft frame with layup C0-N-C0.

Layer Code	First-Order		Second-Order		Third-Order		Fourth-Order	
	F.r (Hz)	*ζ* (%)	F.r (Hz)	*ζ* (%)	F.f (Hz)	*ζ* (%)	F.r (Hz)	*ζ* (%)
C0-C0-C0	49.4	1.567	65.6	1.691	83.8	1.349	94.0	1.715
C0-C60-C0	42.7	1.630	66.5	1.561	83.0	1.375	97.1	1.341
C0-C90-C0	38.9	1.710	66.2	1.671	81.2	1.497	92.3	1.955

**Table 13:** Natural frequency and damping ratio of the raft frame with layup C45-N-C45.

Layer Code	First-Order		Second-Order		Third-Order		Fourth-Order	
	F.r (Hz)	*ζ* (%)	F.r (Hz)	*ζ* (%)	F.f (Hz)	*ζ* (%)	F.r (Hz)	*ζ* (%)
C45-C0-C45	86.5	0.849	109.8	0.662	130.5	1.243	-	-
C45-C60-C45	66.7	1.364	74.7	1.490	122.4	1.508	127.6	1.230
C45-C90-C45	60.2	1.434	66.9	1.468	102.6	1.531	121.0	1.368

**Table 14:** Natural frequency and damping ratio of the raft frame with layup C90-N-C90.

Layer Code	First-Order		Second-Order		Third-Order		Fourth-Order	
	F.r (Hz)	*ζ* (%)	F.r (Hz)	*ζ* (%)	F.f (Hz)	*ζ* (%)	F.r (Hz)	*ζ* (%)
C90-C0-C90	70.0	1.100	70.4	0.999	123.2	1.291	95.3	1.391
C90-C60-C90	50.3	1.573	53.2	1.575	80.8	1.836	85.8	1.423
C90-C90-C90	43.2	1.690	47.4	1.557	76.6	1.566	78.8	1.866

**Table 15:** Layups of the CFRP raft frame components.

Name	Layups	D_11_* (GPa)	D_22_* (GPa)	D_66_* (GPa)	D_16_* (GPa)	D_26_* (GPa)
Top plate	[(90°/0°_2_)_3_/45°/0°/−45°]_S_	86.9	33.7	16.3	2.3	2.3
Bottom plate	[(90°/0°_2_)_3_/45°/0°/−45°]_S_	86.9	33.7	16.3	2.3	2.3
I-support	[(90°/0°_2_)_3_/45°/0°/−45°]_S_	86.9	33.7	16.3	2.3	2.3

**Table 16:** Comparison of natural frequency and damping ratio between test and simulation results of the plates.

Result	Natural Frequency/Hz				Damping Ratio/%			
	First Order	Second Order	Third Order	Fourth Order	First Order	Second Order	Third Order	Fourth Order
Plate 1	17.43	55.02	64.35	73.13	1.399	0.429	0.417	0.402
Plate 2	17.35	55.27	65.22	73.49	1.246	0.496	0.385	0.373
Average of test result	17.39	55.15	64.79	73.31	1.323	0.463	0.401	0.388
Simulation result	20.01	58.18	65.90	77.40	1.155	0.401	0.429	0.332
Error (%)	0.151	0.055	0.017	0.056	−0.13	−0.11	0.07	−0.14

**Table 17:** Comparison of natural frequency and damping ratio of test and simulation results of I-support.

Result	Natural Frequency/Hz				Damping Ratio/%			
	First Order	Second Order	Third Order	Fourth Order	First Order	Second Order	Third Order	Fourth Order
A-1	558.2	-	951.8	1371.8	1.250	-	2.085	0.789
A-2	566.3	-	954.0	1378.6	0.922	-	1.978	0.815
A-3	533.1	-	939.5	1380.0	0.944	-	1.678	0.625
A-4	569.7	-	1059.1	1363.3	1.257	-	1.725	0.807
A-5	567.5	-	1028.0	1382.8	1.093	-	1.546	0.679
A-6	518.0	-	921.8	1299.1	1.095	-	1.658	0.802
Average of test result	552.1	-	975.7	1362.6	1.094	-	1.778	0.802
Simulation result	540.1	676	1012.1	1464.5	1.11	0.51	1.265	0.915
Error (%)	−2.2	-	3.7	7.5	2.1	-	−28.9	14.1

**Table 18:** Modal shape of test and simulation in X/Y direction.

Mode of Vibration	First Order	Second Order	Third Order	Fourth Order
Test result in X direction	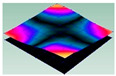	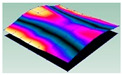	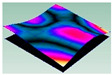	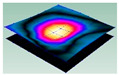
Test result in Y direction	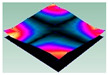	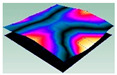	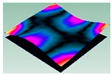	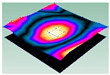
Simulation result in X direction	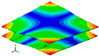	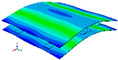	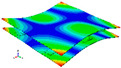	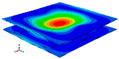
Simulation result in Y direction	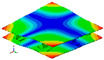	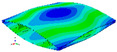	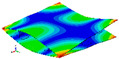	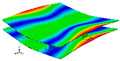

**Table 19:** Comparison of natural frequency and damping ratio of test and simulation results of X/Y direction arrangement.

Result	Natural Frequency/Hz				Damping Ratio/%			
	First Order	Second Order	Third Order	Fourth Order	First Order	Second Order	Third Order	Fourth Order
Test result in X direction	53.8	84.6	99.4	102.3	0.908	0.776	0.445	0.464
Test result in Y direction	54.9	78.4	89.4	97.2	0.512	0.635	0.677	0.541
Simulation result in X direction	75.4	103.2	125.3	133.4	0.711	0.668	0.739	0.687
Simulation result in Y direction	76.4	95.1	118.7	134.5	0.711	0.675	0.800	0.661
Discrepancy of test result (%)	2.3	7.4	10.1	5.0	43.6	18.2	34.3	14.2
Discrepancy of simulation result (%)	1.2	7.9	5.3	0.8	0	1.3	7.6	2.6

## Data Availability

The data presented in this study are available on request from the corresponding author.
